# The Effect of Methamphetamine Use on Radiographic Vasospasm Following Angiogram-Negative Subarachnoid Hemorrhage: A Preliminary Retrospective Analysis

**DOI:** 10.3390/jcm14227921

**Published:** 2025-11-08

**Authors:** Matthew K. McIntyre, Barry Cheaney, Jesse Liu, Aclan Dogan, Olabisi Sanusi

**Affiliations:** Department of Neurological Surgery, Oregon Health & Science University, Portland, OR *97239*, USA

**Keywords:** angiogram-negative subarachnoid hemorrhage, methamphetamine, subarachnoid hemorrhage, outcomes, vasospasm

## Abstract

**Introduction:** The effect of methamphetamine use on angiogram-negative subarachnoid hemorrhage (ANSAH) outcomes has not been examined. Herein, our goal is to evaluate the effect of methamphetamine use on radiographic vasospasm following ANSAH. Methods: This is a retrospective cohort analysis that was performed between 2011 and 2022 among consecutive ANSAH patients presenting to our institution. Methamphetamine positivity (MP) was defined as presence of methamphetamine on urine toxicology and/or by patient report. Outcomes of interest included radiographic vasospasm, clinical vasospasm, and discharge home and were evaluated using multivariate logistic regressions. Results: 101 patients met inclusion criteria and had a median Hunt and Hess score of 2 (range 1–4). Eight (7.9%) MP patients were younger (47.5 ± 3.3 v. 60.8 ± 1.2 years, *p* = 0.004) than methamphetamine-negative patients. In univariate analysis, MP patients were nearly 12 times more likely to experience radiographic vasospasm (odds ratio (OR) 11.6; 95%: confidence interval (CI): 1.4–98.3; *p* = 0.008) but there was no significant difference in clinical vasospasm or discharge home (*p* > 0.05). In multivariate analysis, MP was associated with increased radiographic vasospasm (OR 18.8; 95%CI: 1.7–210.5, *p* = 0.017) but not clinical vasospasm (OR: 5.1; 95%CI: 0.9–28.7; *p* = 0.063) or discharge home (OR: 1.3; 95%CI: 0.1–15.6; *p* = 0.843) Conclusions: Methamphetamine-positive ANSAH patients have increased odds of radiographic vasospasm compared to MN patients. While limited by sample size, this preliminary study adds to our understanding of the increased risk of radiographic vasospasm following ANSAH for patients who use methamphetamine. Future multi-center prospective registry studies should include methamphetamine history as this may modulate vasospasm risk.

## 1. Introduction

Methamphetamine abuse (MA) is a growing public health crisis with usage increasing by 43% between 2015 and 2019, now to over 2 million users in the United States overall [1], and has a high incidence in the western US [2]. Methamphetamine abuse is associated with a 2-fold increased risk for hemorrhagic stroke with a particular burden of disease on younger patients [3]. The mechanism of methamphetamine is via the stimulation of catecholamine release which causes an increased heart rate and blood pressure, which likely leads to the higher incidence of intracranial hemorrhage [3] and has been associated with subarachnoid hemorrhage (SAH) with smaller aneurysms than in non-methamphetamine-using patients [4]. Apart from the well-established increased risk of intracranial hemorrhage, the influence of methamphetamine use on intracranial hemorrhage outcomes remains controversial [5]. Subjectively, SAH patients who present under the influence of methamphetamine appear to have more challenging clinical courses compared to other intoxicants, which could be due to vasospasm [6]. To date, there have only been limited systematic examinations of the effect of methamphetamine use on the cerebral vasculature and clinical outcomes following SAH. Beadell et al. examined the influence of methamphetamine use on aneurysmal SAH (aSAH) outcomes and showed that methamphetamine-positive (MP) patients present with higher-grade aSAH and worse Glasgow outcome scale scores but no difference in vasospasm, length of stay, or cerebral infarction compared to age-matched controls [7].

Subarachnoid hemorrhage (SAH) is a common cause of spontaneous (non-traumatic) intracranial hemorrhage and is associated with high rates of morbidity and mortality. Following the initial physiologic insult of the SAH, patients are typically managed in the intensive care unit for one to three weeks to mitigate the effects of cerebral arterial vasospasm with the goal of preventing delayed cerebral ischemia (stroke). This effort is multimodal and includes frequent neurologic examinations, administration of calcium channel blockers, intensive management of blood pressure and intravascular volume status, and endovascular rescue techniques [8]. For still unknown reasons, only 42% of patients develop vasospasm [9]. Angiogram-negative subarachnoid hemorrhage (ANSAH) represents approximately one fifth of all spontaneous subarachnoid hemorrhage [10,11] and can have a variable clinical course [12] based on the presence of symptomatic vasospasm and hydrocephalus [13]. To date, the influence of methamphetamine use on ANSAH outcomes has not been examined. The goal of this study was therefore to examine the influence of methamphetamine use on vasospasm, complications, and outcomes following ANSAH.

## 2. Methods

### 2.1. Study Design and Setting

This retrospective cohort analysis was conducted at a large tertiary academic center (Oregon Health and Science University) after Institutional review board approval with a waiver of consent (IRB# 00024858, approval date: 14 November 2022). All data was collected using our electronic medical record system between November 2022 and November 2023.

### 2.2. Subject Selection

ANSAH patients were identified using CPT codes (36221-36228) of patients admitted to our institution between 9/1/11 and 8/31/22. Those included were age 18 years at time of hospital admission, presented to our institution with an initial diagnosis of acute radiographically apparent subarachnoid hemorrhage, and had an initial diagnostic of cerebral angiogram-negative for culprit vascular lesion. Patients were excluded if they had a history of cranial trauma within the past 6 months, history of prior subarachnoid hemorrhage, recent stroke including venous infarct, had high initial suspicion for vasculitis or amyloid angiopathy, lack of angiogram, hemorrhagic mass, or primarily intraparenchymal hemorrhage.

### 2.3. Measures and Outcomes

For each patient, the demographics, smoking history, modified frailty index-11, Hunt and Hess (HH) score, World Federation of Neurological Surgeons (WFNSs) score, modified Fisher score, recent methamphetamine usage (as defined by urinalysis or patient report), use of anticoagulation or antiplatelet medication, body mass index (BMI), need for a ventriculostomy (EVD), development of a neurologic deficit during the stay (clinical vasospasm), neurologic exam at discharge, and need for permanent CSF-diverting shunt, were collected. Radiographic vasospasm was defined as the presence of vasospasm to any degree [9] on either CT angiography, MR angiography, or digital subtraction angiography as stated by the attending radiologist or transcranial Doppler Lindegaard/hemispheric ratio >3 during the stay. As defined by prior authors, ANSAH type was divided into either perimesencephalic (PMH-SAH) or non-perimesencephalic (NPAH-SAH) [14,15].

Primary endpoints were discharge home, radiographic vasospasm, and clinical vasospasm. Secondary endpoints were complications, death, radiographic stroke, duration of nicardipine use, need for hypertonic saline, ICU, and hospital length of stay (LOS). A complication was defined as presence of any of the following: stroke requiring thrombectomy, positive blood culture, positive CSF culture, urinary tract infection, vasopressor requirement, ICU bounce-back, groin complication, non-neurosurgical procedure requirement, acute kidney injury (as defined by an abnormal increase in creatine of 0.3 over a 48 h period) [16], seizure, central venous catheter infection, pneumonia, intubation during the admission (outside of a procedure), tracheostomy, gastrostomy, pulmonary embolism, or deep vein thrombosis. Cost analysis was performed using hospital charges.

### 2.4. Statistical Analysis

A student *t*-test [17] was used for continuous variables and data are shown using mean ± standard error of the mean (SEM) after normality was established using Anderson–Darling tests and homogeneity was confirmed with a Levene’s test of equality of variances. A fisher’s exact test of independence [18] was used for binary variables and odds ratios (OR) are shown with 95% confidence intervals (CI) after calculation in a 2 × 2 table. Odds ratios are shown in reference to the MN-negative group. Given the change in practice from Fisher score to modified Fisher score at our institution, records of those who had Fisher scores recorded were examined and a modified Fisher score was calculated. Other missing data were omitted from analysis. To evaluate the independent effect of methamphetamine use on outcomes, multivariate logistic regressions [19] were performed for primary endpoints and controlled for age, sex, HH score, and modified Fisher score as these are known biologically relevant factors to influence primary endpoints. No co-linearity was detected as defined previously as a variance inflation factor of less than 1 or greater than 10 [15]. Statistical analysis was performed using SPSS version 29 (IBM corp., Armonk, NY) and significance was defined as an alpha < 0.05.

## 3. Results

**Figure 1:**
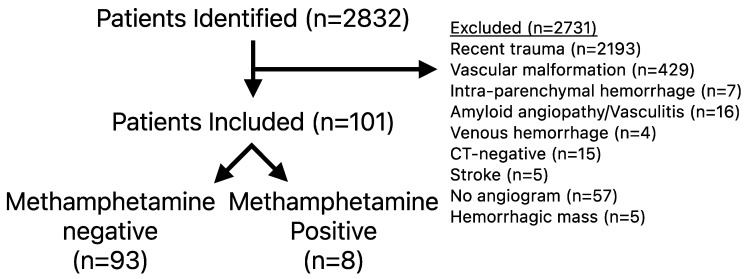
Inclusion and exclusion criteria.

Of the 2831 patients screened, 101 met inclusion and exclusion criteria (Figure 1). For those who met inclusion criteria, the average age was 59.7 ± 1.2 years, the median Hunt and Hess score was 2 (range 1–4), WFNSs score of 1 (range 1–5), modified Fisher score of 3 (Range 1–4), and 51 had perimesencephalic distribution (51/101, 50.5%) (Table 1). A total of 8 (7.9%) patients were identified who had recent methamphetamine use (MP) and their clinical course is summarized in Table 2. MP patients were younger (47.5 ± 3.3 versus 60.8 ± 1.2 years, *p* = 0.004) but had no difference in smoking history (*p* = 0.068), ANSAH type (*p* = 0.160), Hunt and Hess score (*p* = 0.585), modified Fisher score (*p* = 0.846), baseline frailty status (*p* = 0.674), or requiring a ventriculostomy (*p* = 0.470) (Table 1).

Methamphetamine-positive (MP) patients had nearly twelve times increased odds of developing radiographic vasospasm (OR 11.6; 95%: 1.4–98.3, *p* = 0.008) but no significant difference in clinical vasospasm (*p* = 0.233) or discharge home (*p* = 0.678) rates (Table 3). MP patients had higher rates of hypertonic saline use (OR 8.50 95%CI: 1.01–71.9, *p* = 0.028) but no significant difference in need for intra-arterial verapamil (*p* = 0.511), stroke (*p* = 0.233), or days requiring nicardipine (*p* = 0.879). None of the MP patients had a subsequent angiogram showing an aneurysmal source or known repeat SAH. One patient in the MP group died; however, this was non-significant compared to MN patients (*p* = 0.344). There were no significant differences in complications, hospital or ICU length of stay, or hospital charges between MP and MN patients (Table 3 and Table 4).

To investigate the effect of methamphetamine-use on primary endpoints, multivariate logistic regressions were performed. When controlling for age, sex, Hunt and Hess score, and modified Fisher score, methamphetamine use was independently associated with increased radiographic vasospasm (odds ratio (OR): 18.8; 95%CI: 1.7–210.5, *p* = 0.017) but not clinical vasospasm (OR: 5.1; 95%CI: 0.9–28.7; *p* = 0.063) or discharge home (OR: 1.3; 95%CI: 0.1–15.6; *p* = 0.843) (Table 5).

## 4. Discussion

Methamphetamine (MA) is a powerful sympathomimetic that is associated with an elevated risk of subarachnoid hemorrhage. The effect of MA on ANSAH outcomes has yet to be evaluated and, in this study, we show that MP ANSAH patients have higher rates of radiographic but not clinical vasospasm and no difference in short-term patient outcomes.

Prior work examining methamphetamine use in subarachnoid hemorrhage has focused on aneurysmal SAH, often finding that MP is associated with poorer outcomes. Baedell et al. found among aneurysmal subarachnoid hemorrhage patients that MA was associated with higher-grade hemorrhage and poorer Glasgow outcome scale scores but no difference in length of stay or cerebral infarction compared to age-matched controls. In univariate analysis, these authors also showed that MP patients had significantly higher rates of vasospasm but when age-matched or adjusted in multivariate analysis, there was no effect of MA on vasospasm rates [7]. Likewise, Moon et al. showed among 472 patients of the Barrow Ruptured Aneurysm trial that recent MA use was an independent predictor of poor Glasgow outcome score at 1 and 3 years but found no difference in vasospasm or hemorrhage grade [20]. Baedell defined radiographic vasospasm by both angiography and transcranial Doppler while the Moon study by only angiography. We sought to be highly sensitive for vasospasm and included any imaging showing vasospasm including CT or MRI angiography, digital subtraction angiography or TCD. We found similar rates of vasospasm compared to prior studies of ANSAH patients [15]. It is also worth noting that in both of these prior studies in addition to our own, the effect of socioeconomic influences on long-term outcomes was not thoroughly investigated, will likely influence outcome scores in this patient population, and should be included in future research.

Our study found that when controlling for age and admission subarachnoid hemorrhage-grade, MA use was independently associated with radiographic vasospasm. This observation was also supported by the increased hypertonic saline requirement of MP patients as hyponatremia is a finding often associated with microvascular vasospasm and increased delayed cerebral ischemia [21]. However, the clinical significance of this is unclear given the lack of difference between groups in symptomatic vasospasm, discharge home, or neurologic deficit at discharge but we did observe a non-significant trend toward increased stroke (OR 2.58; 95%CI: 0.60–11.1). Together, these results suggest that it continues to be prudent to monitor MP patients closely for signs of vasospasm and hyponatremia.

Given the heterogenous clinical course of ANSAH patients, recent efforts have focused on patient risk stratification to identify a subset of patients that can be safely managed with shorter hospital and intensive care unit length of stays [13]. In particular, Alrohimi et al. showed that ANSAH patients without hydrocephalus may represent a more benign patient population [13]. In addition to hydrocephalus, the results of this paper suggest that patients with recent methamphetamine use may represent a higher risk patient population and should be monitored throughout the vasospasm window.

The mechanism of ANSAH is not well understood and it is unclear what role methamphetamine use plays in the development of ANSAH. Ho et al. postulated that MA use is associated with ischemic stroke because of vasculitis, thereby resulting in reduced cerebral vessel caliber via an inflammatory mechanism. On a long-term basis, some have argued that this prolonged MA exposure causes a chronic hypertension resulting in premature atherosclerosis which puts patients at risk for all-cause stroke [22,23]. It is likely that methamphetamine-induced hypertension likely plays a role in ANSAH development but this was not examined in this study. In our cohort, we found no difference in the duration of nicardipine requirement, which might be due to the small size of MA + patients or related to other factors such as the short duration or action, and the chronicity of methamphetamine abuse. Furthermore, the MA-associated premature atherosclerosis could explain the trend toward increased stroke rates but this is difficult to study in this patient population given the rarity of the disease. Interestingly, as with MN patients, none of the MP patients were known to have had a repeat SAH or found to have an aneurysm on later imaging. This is information that can be valuable in coaching patients that once recovered from ANSAH, as we can expect that a repeat hemorrhage would be very rare. Finally, given the retrospective nature of this study and the frequent loss to follow-up after discharge, we are unable to evaluate the long-term influence of methamphetamine use on outcomes after ANSAH, which could be a future line of research.

### Limitations

In addition to its retrospective design, the major limitation of this study is the rarity of ANSAH and methamphetamine use. We examined 101 patients over an 11-year period finding only 8 who had reported MA use. It is our protocol for all patients to receive a urine toxicology screen on admission and therefore the risk of reporting bias is low. The rate of MA use was similar to the Baedell study (7% of aSAH patients) at our institution 11 years prior [7]. This sample size is the maximum that was reasonably achievable based on available medical records; however, the risk of type II error and multivariate modeling overfitting remains present. As alluded to earlier, the second limitation of this study is lack of long-term follow-up and while all but one MP patient was discharged home, the socioeconomic influence that methamphetamine use has on our outcomes and poor follow-up should not be overlooked. Third, given the lack of consensus on ‘radiographic vasospasm [9],’ we defined it by any degree of vasospasm on the attending radiologist read of vasospasm on any vascular imaging as a binary endpoint rather than as a percentage change in vessel narrowing, which could have introduced variability in our primary endpoint.

## 5. Conclusions

Angiogram-negative subarachnoid hemorrhage has a variable clinical course. Methamphetamine use is a growing public health crisis in our region. We found that MP patients who present with ANSAH have increased rates of radiographic vasospasm. While limited by sample size, this preliminary study adds to our understanding of the increased risk of radiographic vasospasm following ANSAH for patients who use methamphetamine. Future multi-center prospective registry studies should include methamphetamine history as this may modulate vasospasm risk.

## Figures and Tables

**Table 1:** Baseline characteristics of ANSAH patients stratified by methamphetamine (MA) use. Odds ratios (OR) with 95% confidence intervals are shown when able to be calculated and significant *p*-values are bolded. Odds ratios are shown in reference to the MA-negative group.

	Overall *(n* = 101) Mean ± Standard Error of the Mean (SEM) or Number (%)	MA-Negative (*n* = 93)	MA-Positive (*n* = 8)	OR (95% CI)	*p*-Value
Age (years)	59.7 ± 1.2	60.8 ± 1.2	47.5 ± 3.3	-	0.004
Male	59 (58.4%)	55 (59%)	4 (50%)	0.7 (0.2–2.9)	0.716
Ever smoker	55 (55%)	47 (52%)	7 (88%)	6.6 (0.8–55.5)	0.068
Perimesencephalic hemorrhage distribution	51 (51%)	49 (53%)	2 (25%)	0.30 (0.06–1.56)	0.160
Hunt and Hess score	2.0 ± 0.07	2.0 ± 0.8	1.9 ± 0.2	-	0.585
Modified Fisher Score	2.5 ± 0.1	2.5 ± 0.1	2.6 ± 0.4	-	0.846
World Federation of Neurological Surgeons score	1.5 ± 0.1	1.6 ± 0.1	1.3 ± 0.2	-	0.149
Body mass index (kg/m^2^)	30.16 ± 2.08	29.9 ± 2.2	33.4 ± 3.9	-	0.446
Frailty (mFI-11)	1.37 ± 0.14	1.4 ± 0.2	1.3 ± 0.3	-	0.674
Angiogram with unrelated/unruptured lesion	13 (13%)	12 (13%)	1 (13%)	0.96 (0.11–8.54)	>0.999
Required ventriculostomy	38 (38%)	34 (37%)	4 (50%)	1.74 (0.41–7.39)	0.470
Neurologically intact on arrival	68 (67%)	62 (67%)	6 (75%)	1.50 (0.29–7.87)	>0.999
Anti-thrombotic use	8 (8%)	8 (8.6%)	0 (0.0%)	-	>0.999
Aspirin use	27 (27%)	26 (28%)	1 (13%)	0.37 (0.04–3.14)	0.678

**Table 2:** Characteristics and clinical course of Methamphetamine-positive patients. ANSAH type was defined as either perimesencephalic (PMH) or non-perimesencephalic (NPH) ANSAH.

Age	Sex	Current Smoker	ANSAH Type	Modified Fisher Score	Hunt and Hess Score	Radiographic Vasospasm	Clinical Vasospasm	Intact on Discharge	Hypertonic Saline Use	Need for Shunt	Inpatient Length of Stay in Days	Days of Nicardipine	Stroke	Discharge Destination
56	Male	Yes	NPH	2	2	Yes	Yes	Yes	Yes	No	12	2	Yes	Home
57	Male	Yes	NPH	1	2	Yes	Yes	No	Yes	No	15	2	Yes	Death
50	Male	Yes	NPH	1	2	Yes	No	Yes	Yes	Yes	14	0	No	Home
38	Female	Yes	NPH	4	3	Yes	Yes	Yes	Yes	Yes	12	1	No	Home
49	Male	Yes	PMH	1	1	Yes	No	Yes	Yes	No	11	1	Yes	Home
54	Female	No	NPH	1	1	Yes	Yes	Yes	Yes	No	13	1	Yes	Home
30	Female	Yes	NPH	1	2	No	No	Yes	No	No	7	1	No	Home
46	Female	Yes	PMH	3	2	Yes	No	Yes	Yes	No	11	2	No	Home

**Table 3:** Univariate analysis of primary and secondary outcomes stratified by methamphetamine use. Odds ratios (OR) with 95% confidence intervals are shown when able to be calculated and significant *p*-values are bolded. Odds ratios are shown in reference to the MN-negative group.

	Overall (*n* = 101) Mean ± SEM or Number (%)	Meth-Negative (*n* = 93)	Meth-Positive (*n* = 8)	OR (95% CI)	*p*-Value
Radiographic vasospasm	42 (41.6%)	35 (38%)	7 (88%)	11.6 (1.4–98.3)	0.008
Neurologic deficit during stay (clinical vasospasm)	30 (30%)	26 (28%)	4 (50%)	2.58 (0.60–11.1)	0.233
Discharge home	78 (77%)	71 (77%)	7 (88%)	2.17 (0.25–18.6)	0.678
Repeat angiogram obtained	87 (86%)	79 (85%)	8 (100%)	-	0.595
Repeat angiogram positive for culprit lesion	2 (2%)	2 (2.5%)	0 (0%)	-	>0.999
Intra-arterial verapamil given	8 (8%)	7 (7.9%)	1 (13%)	1.67 (0.18–15.6)	0.511
Stroke on any imaging	30 (30%)	26 (28%)	4 (50%)	2.58 (0.60–11.1)	0.233
Death within 30 days	5 (5%)	4 (4%)	1 (13%)	3.18 (0.31–32.4)	0.344
Death prior to discharge	3 (3%)	2 (2%)	1 (13%)	6.50 (0.15–0.52)	0.221
Neurologically intact on discharge	86 (85%)	79 (85%)	7 (88%)	1.24 (0.141–10.9)	>0.999
Days requiring nicardipine	1.6 ± 0.2	1.6 ± 0.2	1.5 ± 0.4	-	0.879
Received hypertonic saline	49 (49%)	42 (45%)	7 (88%)	8.50 (1.01–71.9)	0.028
Need for shunting	9 (9%)	7 (8%)	2 (25%)	4.10 (0.69–24.2)	0.149
ICU length of stay (days)	9.7 ± 0.5	9.7 ± 0.6	10.0 ± 0.9	-	0.756
Ward length of stay (days)	2.4 ± 5.2	2.5 ± 0.5	1.1 ± 0.5	-	0.074
Hospital length of stay (days)	12.0 ± 7.4	12.0 ± 0.8	11.9 ± 0.9	-	0.945
Total Cost ($)	192,615 ± 7493	190,807 ± 8052	213,419 ± 12,886	-	0.416

**Table 4:** Complications stratified by methamphetamine use. Odds ratios (OR) with 95% confidence intervals are shown when able to be calculated and significant *p*-values are bolded. Odds ratios are shown in reference to the MN group.

	Overall (*n* = 101) Mean ±SEM or Number (%)	Meth-Negative (*n* = 93)	Meth-Positive (*n* = 8)	OR (95% CI)	*p*-Value
Any complication	31(31%)	27 (29%)	4 (50%)	2.4 (0.6–10.5)	0.245
Stroke requiring thrombectomy	2 (2%)	2 (2.2%)	0 (0.0%)	-	>0.999
Positive blood culture	3 (3%)	3 (3.2%)	0 (0.0%)	-	>0.999
Positive cerebrospinal fluid culture	2 (2%)	2 (2.2%)	0 (0.0%)	-	>0.999
Urinary tract infection	17 (17%)	15 (16%)	2 (25%)	1.7 (0.3–9.4)	0.619
Vasopressor use	6 (6%)	5 (5.4%)	1 (13%)	2.5 (0.3–25.6)	0.399
ICU bounce-back	1 (1%)	1 (1.1%)	0 (0.0%)	-	>0.999
Groin complication	0 (0%)	0 (0%)	0 (0%)	-	>0.999
Non-neurosurgical surgery (excluding tracheostomy/gastrostomy)	0 (0%)	0 (0%)	0 (0%)	-	>0.999
Acute kidney injury	13 (13%)	13 (14%)	0 (0.0%)	-	0.592
Clinical seizure	4 (4%)	3 (3.2%)	1 (13%)	4.3 (0.4–47)	0.285
Central line infection	0 (0%)	0 (0%)	0 (0%)	-	>0.999
Pneumonia	4 (4%)	3 (3.2%)	1 (13%)	4.3 (0.4–47)	0.285
Intubation during admission	12 (12%)	10 (11%)	2 (25%)	2.8 (0.5–16)	0.241
Gastrostomy tube	1 (1%)	1 (1.1%)	0 (0%)	-	>0.999
Tracheostomy tube	1 (1%)	1 (1.1%)	0 (0%)	-	>0.999
Pulmonary embolism	1 (1%)	1 (1.1%)	0 (0%)	-	>0.999
Deep vein thrombosis	3 (3%)	2 (2.2%)	1 (13%)	6.5 (0.52–81)	0.221

**Table 5:** Multivariate analysis for each of the primary endpoints. *p*-values are shown and bolded if significant. OR: odds ratio, CI: confidence interval.

	Radiographic Vasospasm		Clinical Vasospasm		Discharge Home	
	OR (95%CI)	*p*-Value	OR (95%CI)	*p*-Value	OR (95%CI)	*p*-Value
Age	1.0 (0.9–1.0)	0.126	1.0 (1.0–1.1)	0.186	0.9 (0.9–1.0)	0.051
Sex	2.8 (1.0–7.6)	0.045	1.3 (0.5–3.7)	0.618	0.5 (0.1–2.0)	0.345
Hunt and Hess Score						
Grade 1	Reference	0.040	Reference	0.005	Reference	0.002
Grade 2	3.3 (0.8–14.4)	0.110	1.6 (0.4–6.3)	0.505	0.3 (0.05–2.0)	0.224
Grade 3	15.6 (1.8–137.1)	0.013	15.2 (2.1–110.4)	0.007	0.02 (0.002–0.2)	0.003
Grade 4	14.7 (1.4–150.4)	0.023	25.0 (2.1–294.0)	0.010	0.01 (0.001–0.2)	0.003
Modified Fisher Score						
Grade 1	Reference	0.139	Reference	0.945	Reference	0.099
Grade 2	1.1 (0.3–4.6)	0.885	1.4 (0.3–6.9)	0.680	4.2 (0.3–64.6)	0.307
Grade 3	0.2 (0.05–0.9)	0.031	1.5 (0.4–5.9)	0.560	0.2 (0.05–1.1)	0.071
Grade 4	0.7 (0.2–2.4)	0.589	1.3 (0.3–5.1)	0.698	0.9 (0.2–5.3)	0.949
Methamphetamine Use	18.8 (1.7–210.5)	0.017	5.1 (0.9–28.7)	0.063	1.3 (0.1–15.6)	0.843

## Data Availability

Data can be made available upon reasonable written request. The original contributions presented in this study are included in the article. Further inquiries can be directed to the corresponding author.
